# Severity in a Norwegian hospitalized injury material (N = 177,663) by two severity measures: threat-to-life and threat of disability: Johan Lund

**DOI:** 10.1093/eurpub/ckac129.632

**Published:** 2022-10-25

**Authors:** C Madsen, J Lund

**Affiliations:** National Public Health Institute, Oslo, Norway

## Abstract

We conducted a registry-based cohort study of all individuals aged 25-64 years residing in Norway by 1st of January 2008 (N = 2,535,213). This cohort was followed from 2008 through 2014 using inpatient registrations for acute hospitalizations due to all-cause injuries, getting 177,663 cases of hospitalized persons (incidence rate of new cases: 102.1 pr 10,000 person-years at risk). We derived two measures of severity: threat-to-life using the International Classification of Disease-based Injury Severity Score (ICISS) (Stephenson et al 2004; Gedeborg et al 2014), and threat of disability using long-term disability weights (DW) from the Injury-VIBES project (Gabbe et al 2014), also based on the ICD-codes. We found the following distributions of the hospitalized persons (N = 177,663): 1) Threat to life (ICISS): High threat to life: 4,186 (2.4 %); Lower threat to life: 173,477 (97.6%). 2) Threat of disability (Injury Vibes DW): High probability of long-term disability (DW-score < 0.807): 36,573 (20,6 %); Medium probability of long-term disability (DW-score 0.807-0.947): 97,590 (54,9 %); low probability of long-term disability (DW-score >0.947): 43,530 (24.5 %). Correlation between ICISS-score and the Injury Vibes disability weight score was moderate (r = 0.418, p < 0.001). The presentation will end up in a discussion on which of these two severity measures could be used for comparing burden of injuries across countries.

References

Gabbe BJ, Simpson PM, Lyons RA, et al. (2014) Association between the Number of Injuries Sustained and 12-Months Disability Outcomes: Evidence from the Injury-VIBES Study. PLos ONE, 9(12):e113467. DOI: 10.1371/journal.pone.0113467.

Gedeborg R, Warner M, Chen LH, et al. (2014) Internationally comparable diagnosis-specific survival probabilites for calculation of the ICD-10-based Injury Severity Score. J Trauma Acute Care Surg, 76(2):358-65. DOI: 10.1097/ta.0b013e3182a9cd31.

